# Deep semi-supervised learning ensemble framework for classifying co-mentions of human proteins and phenotypes

**DOI:** 10.1186/s12859-021-04421-z

**Published:** 2021-10-16

**Authors:** Morteza Pourreza Shahri, Indika Kahanda

**Affiliations:** 1 Gianforte School of Computing, Montana State University, Bozeman, USA; 2 School of Computing, University of North Florida, Jacksonville, USA

**Keywords:** Biomedical relationship extraction, Protein phenotype relationships, Human phenotype ontology, Semi-supervised learning, Ensemble learning, Deep learning

## Abstract

**Background:**

Identifying human protein-phenotype relationships has attracted researchers in bioinformatics and biomedical natural language processing due to its importance in uncovering rare and complex diseases. Since experimental validation of protein-phenotype associations is prohibitive, automated tools capable of accurately extracting these associations from the biomedical text are in high demand. However, while the manual annotation of protein-phenotype co-mentions required for training such models is highly resource-consuming, extracting millions of unlabeled co-mentions is straightforward.

**Results:**

In this study, we propose a novel deep semi-supervised ensemble framework that combines deep neural networks, semi-supervised, and ensemble learning for classifying human protein-phenotype co-mentions with the help of unlabeled data. This framework allows the ability to incorporate an extensive collection of unlabeled sentence-level co-mentions of human proteins and phenotypes with a small labeled dataset to enhance overall performance. We develop PPPredSS, a prototype of our proposed semi-supervised framework that combines sophisticated language models, convolutional networks, and recurrent networks. Our experimental results demonstrate that the proposed approach provides a new state-of-the-art performance in classifying human protein-phenotype co-mentions by outperforming other supervised and semi-supervised counterparts. Furthermore, we highlight the utility of PPPredSS in powering a curation assistant system through case studies involving a group of biologists.

**Conclusions:**

This article presents a novel approach for human protein-phenotype co-mention classification based on deep, semi-supervised, and ensemble learning. The insights and findings from this work have implications for biomedical researchers, biocurators, and the text mining community working on biomedical relationship extraction.

## Background

Proteins perform a wide range of operations in cells, and they are considered the workhorses of life. The cooperation of thousands of proteins provides the functionality of cells. However, genetic sequence errors of proteins may cause alterations in the protein’s structure. These may lead to a change in the protein’s function-specific structure, resulting in phenotype alterations [1]. Medically, a phenotype is a deviation from normal morphology or physiology [2]. Typically, the genotype-phenotype correlations are very challenging to interpret due to the scarcity of genomic variants that cause rare diseases [3]. Therefore, one way to increase certainty is to identify the patients who have the same phenotype characteristics and share the same or overlapping gene variants [3]. Overall, finding the relationships between proteins and phenotypes is essential for downstream applications, e.g., finding the treatments for rare diseases.

Human Phenotype Ontology, also known as HPO, is a standard and structured vocabulary of phenotypic abnormalities observed in human diseases [4]. HPO comprises of five sub-ontologies including, *Phenotypic abnormalities*, *Mode of inheritance*, *Clinical modifier*, *Clinical course*, and *Frequency*. Clinical abnormalities are described in *Phenotypic abnormalities*, which is the main sub-ontology. It provides HPO terms with their unique HPO Identifiers (IDs), such as *Parkinsonism* (HP:0001300). HPO is structured as a Directed Acyclic Graph (DAG) in which the depth of a term correlates to how specific it is. The *is-a* relationships are also available for each parent-child pair. For the rest of this paper, we use the terms “*HPO term*” and “*phenotype*” interchangeably. Gold-standard annotations for an extensive collection of proteins are maintained in the official HPO website.^1^ Yet, the expansion of HPO annotations over time shows that the HPO database is still incomplete and requires more effort [4–6]. HPO database currently maintains annotations for a little of over 4,500 unique genes.

Gold-standard HPO annotations are typically acquired from biomedical literature using biocuration, which extracts knowledge from unstructured text and stores the data in knowledge bases. In general, biocuration is considered resource-consuming and tedious, often manually performed with some assistance from text mining tools. Hence, for quickly extending knowledge bases, biocurators require accurate computational tools to expedite their curation efforts [7]. Consequently, developing text mining tools to extract protein-phenotype relationships has attracted researchers working in biomedical natural language processing [8–13].

**Fig. 1:**
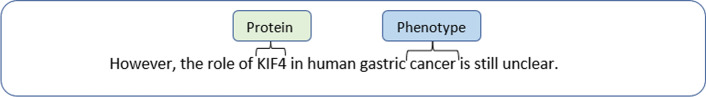
A *bad* (i.e. invalid) co-mention of the protein (“KIF4”) and the phenotype “cancer” extracted from the article PMID: 20711700. Here, the sentence by itself does not bear a meaningful relationship between the two entities

Recently [14], we presented a two-step novel approach capable of extracting the relationships between protein-phenotype terms from biomedical literature. In the first step, we identify the co-occurrences of proteins and phenotypes from abstracts and open access full-text articles from Medline and PubMed Central (PMC) using an advanced text mining pipeline developed by our lab [15]. Then, we extract co-occurrences at various levels concerning the particular span of text from which they are extracted: sentence-level, paragraph-level, and document-level. We refer to these co-occurrences as protein-HPO term *co-mentions*. These co-mentions are currently maintained in ProPheno,^2^ a dataset that maintains records of occurrences of proteins and phenotypes (HPO terms), which is publicly available online [16]. The ProPheno dataset covers *Phenotypic abnormality* sub-ontology. Previously, we showed that these co-mentions are useful in the task of protein-phenotype prediction [15]. However, protein-phenotype co-mentions may or may not convey an actual biological relationship between the two entities (Fig. 1 provides an example of an invalid co-mention).

**Fig. 2:**
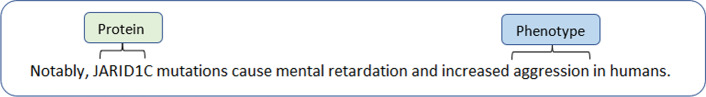
A *good* (i.e. valid) sentence-level co-mention found in the article PMID:18596936

In the second step, we develop a co-mention classifier for distinguishing good versus bad co-mentions. If the context surrounding the entities contains enough evidence supporting a valid relationship, it is referred to as a *good* co-mention. Figure 2 depicts such a good co-mention. Hence, our previously proposed protein-phenotype relation extraction pipeline is a combination of a co-mention extractor and classifier. While the process of extracting co-mentions is demanding due to its resource-consuming nature, developing an accurate co-mention classifier is relatively more challenging.

In our preliminary attempts at formulating a co-mention classifier, we developed 1) PPPred [14], which uses Support Vector Machines (SVMs) and a large collection of semantic and syntactic features, and 2) DeepPPPred [17], an extended version of PPPred, which utilizes an ensemble of PPPred and deep neural networks. We randomly selected a relatively small subset of sentence-level co-mentions stored in the ProPheno database and then curated them with biologists’ assistance. This final gold-standard dataset comprised of 1685 co-mentions.^3^

While both of the aforementioned supervised classifiers (i.e., PPPred and DeepPPPred) were effective at outperforming baseline methods, we observed that their performances plateaued due to the scarcity of the labeled data [14]. We considered two potential solutions to this problem: 1) manually annotating more co-mentions, 2) taking advantage of unlabeled co-mentions without manual intervention. As stated earlier, manual annotation of data is highly resource consuming, and hence annotating more data was not a feasible solution for our task. However, millions of unlabeled protein-phenotype co-mentions are available through the ProPheno [16], which could be utilized for improved performance with a semi-supervised learning setup. In fact, for many of the entity pairs, we have access to at least several sentences, such as the following examples (entities are underlined):

- “*BRCA1*, BRCA2, PALB2 and RAD51C should be included in the genetic testing panel of *breast cancer* patients in Argentina.” (PubMedID: 31446535)
- “Population frequencies of pathogenic alleles of *BRCA1* and BRCA2: analysis of 173 Danish *breast cancer* pedigrees using the BOADICEA model.” (PubMedID: 31435815)

In this work, we study the problem of developing a more effective co-mention classifier by incorporating unlabeled data. More specifically, we describe a novel framework for co-mention classification that combines the advantages of deep learning, semi-supervised learning, and ensemble learning. Our proposed deep semi-supervised ensemble framework for relation extraction requires only a small labeled dataset, to begin with. Furthermore, we develop a prototype of our framework by instantiating it using a self-trained BERT [18] (Bidirectional Encoder Representations from Transformers) classifier combined with an ensemble model composed of convolutional neural networks (CNN) and recurrent neural networks (RNN). We name this prototype PPPredSS (Protein-Phenotype Predictor Semi-Supervised). Using the above-mentioned curated dataset of protein-phenotype sentence-level co-mentions, we demonstrate that PPPredSS provides state-of-the-art performance in human protein-phenotype co-mention classification. PPPredSS outperforms PPPred, DeepPPPred, and S3VM [19] (state-of-the-art SVM for semi-supervised learning). Also, we conduct a use-case study in which we inquire a group of biologists to evaluate the quality of PPPredSS retrieved sentences. The findings from this survey further highlight the utility of our approach. Our software repositories are made publicly available for the benefit of interested researchers.^4^

### Related work

We categorize the existing biomedical relation extraction methods into three main categories: (1) co-occurrence-based methods, (2) rule-based methods, and (3) machine learning-based methods. Co-occurrence methods are the most straightforward technique for extracting the relationships between the entities of interest. They look for any co-occurrence of the two entities in a specific short span of text. These methods typically achieve lower precision yet higher recall values [20]. On the other hand, Rule-based methods extract the relationships using pre-defined linguistic patterns [21–25]. One or more subject matter experts typically provide these rules/patterns. Finally, Machine learning-based methods are also popular for biomedical relation extraction [11, 13, 26, 27]. Various studies discuss supervised and unsupervised methods and show improvement in various biomedical relation extraction tasks [9, 28–30].

Biomedical relation extraction has widely utilized deep learning in various studies [31–37]. Some researchers have created hybrid models by combining RNNs and CNNs [12, 17, 38]. For example, an ensemble composed of RNNs, CNNs, and SVMs, are introduced by Peng et al. [12] to solve BioCreative VI’s chemical-protein relation extraction task.^5^ However, deep neural networks typically are data-hungry. BERT, a pre-trained language representation based on bi-directional transformers, provides a solution to this problem by requiring only a relatively small labeled dataset. Since BERT comes pre-trained on large corpora of text, it only requires fine-tuning of its pre-trained parameters for a given task.

Several studies employ semi-supervised learning using neural networks [39, 40]. For instance, Lin et al. utilize self-training with neural networks for temporal relation extraction tasks, which achieves a new state-of-the-art performance on Clinical TempEval 2017 Task [41]. Khordad and Mercer present a model for extracting the genotype-phenotype relations, which employs a self-supervised approach for enlarging the training set [11].

Deep neural networks, with the help of self-training, can overcome noisy labels without additional supervision [42]. They are also instrumental in ensemble learning settings. Some of the best relation extraction methods, such as for extracting chemical-protein relations, use ensemble learning [12, 38]. Ensemble classifiers have several advantages: (1) Their general performance is higher than their constituent classifiers. (2) They offer a convenient method to combine several models bypassing the need for model selection [43].

Besides text mining methods, several other approaches use gene expression data and network-based models. For instance, Ren et al. present a similarity network for phenotype ontology, followed by network analysis methods for discovering phenotype/disease clusters [44]. Subsequently, they perform the prediction of protein-phenotype associations using machine learning. Zhang et al. [45] employ advanced feature selection methods: Monte Carlo feature selection (MCFS) and incremental feature selection (IFS) for biomarker selection followed by an SVM classifier. Other similar studies utilize Gene Ontology (GO) [46] and KEGG pathways [47, 48], a network embedding algorithm (i.e., node2vec) [49] for discovering disease-related genes and a Convolutional Neural Network for the identification of cell cycle-regulated genes [50]. The random walk with restart algorithm and Laplacian heat diffusion are also extensively studied for gene expression and detection of disease-related genes [51–53].

Despite considerable recent progress on relationships extraction (including a few methods that can extract gene-phenotype relationships), only two methods are explicitly designed for extracting the relationships between human proteins and HPO terms directly from biomedical literature. They are (1) PPPred [14], and (2) DeepPPPred [17], previously developed by our lab. Hence, we use these two previously developed methods as comparators for evaluating the proposed deep semi-supervised ensemble model for co-mention classification. While there are other methods for predicting HPO terms for a given protein using heterogeneous data sources such as PHENOstruct [15, 54], Notaro et al. [55], HPO2Protein [56], AiProAnnotator [57], DeepPheno [58], HPOLabeler [59], HPOAnnotator [60], and HPOFiller [61], they do not employ any text-mining techniques to directly extract relations from biomedical literature. Therefore, these methods are not directly comparable to our proposed model.

## Results and discussion

### Supervised learning component of PPPredSS

In our proposed framework, we required an accurate supervised model to make predictions on the unlabeled instances. We compared several models to select the most accurate model for predictions. We trained them on the training set and evaluated them on the validation set to evaluate the models. Table 1 shows the results of this comparison. The highest obtained Precision, Recall, F1, and AUROC values are bolded in the table.

**Table 1:** Comparison of the performance of various supervised models trained on the training set and evaluated on the validation set

Method	Precision	Recall	F1	AUROC	SD
PPPred	0.741	0.89	0.809	0.657	N/A
RNN	**0.75**	0.795	0.772	0.651	0.006
CNN	0.732	0.763	0.747	0.623	0.008
CNN & RNN	0.732	0.822	0.774	0.658	0.005
BERT	0.745	**0.936**	**0.83**	**0.671**	0.005

CNN & RNN is the model using the average prediction probabilities of the individual CNN and RNN models

BERT model achieved the best performance on the validation set. So, we used that as our primary model for making predictions on the unlabeled instances. Note that in this comparison, we excluded DeepPPPred [17] since it has a relatively long training time, and it is not feasible to be used for making predictions on millions of unlabeled sentences. Another observation is that CNNs and RNNs perform relatively worse on the validation set. This reduction in performance may be because deep neural networks using CNNs and RNNs require a lot of data to be trained well compared to a BERT model. However, combining the predictions of CNNs and RNNs achieves better performance than the individual CNN model and the RNN model, leading to the ensemble of two models performing better than its constituent models.

### Semi-supervised learning component of PPPredSS

In our method, the supervised learning model is used to make predictions on unlabeled data. Then a randomly selected set of top predictions are added to the training data for its expansion. To determine the best size of added unlabeled instances to the training set, we performed experiments with the BERT model and various sizes of 1000, 2000, 3000, 5000, and 10,000 instances. We are reporting the average performance of 10 executions. According to F1 scores reported in Table 2, we select 5000 as the default value for the size of added training instances.

**Table 2:** performance using various sizes of added training examples to the original training set

	Added training size				
	1000	2000	3000	5000	10000
Precision	0.774	0.78	0.776	0.78	**0.782**
Recall	0.864	0.87	0.876	**0.882**	0.864
F1	0.804	0.81	0.82	**0.829**	0.824
AUROC	0.664	0.69	0.704	**0.712**	0.71
SD	0.004	0	0.006	0.006	0.004

The models have been trained on several subsets with various sizes of unlabeled data combined with our training set, and the evaluation is performed on the validation set

### Overall performance of PPPredSS versus others

Table 3 provides a comparison between our proposed deep semi-supervised ensemble model with S3VM, which is the state-of-the-art semi-supervised model based on SVMs [19]. We ran S3VM with features introduced in PPPred study [14] and TFIDF (term frequency-inverse document frequency) features. We used the hyperparameter values recommended by its authors for text data [19]. We fed our training set and all the unlabeled co-mentions into S3VM as input. This approach ensured that S3VM has access to the same data as PPPredSS. Furthermore, we compared our proposed model with PPPred [14] and DeepPPPred [17]. We observed that our proposed model outperformed its comparators significantly, suggesting that the addition of unlabeled co-mentions is beneficial for improved performance.

**Table 3:** Comparison of our proposed model (PPPredSS) versus semi-supervised SVM (S3VM), PPPred, and DeepPPPred

Method	Precision	Recall	F1	AUROC	*p* Value
PPPred	0.898	0.906	0.902	0.845	3.7E-4
DeepPPPred	0.871	**0.973**	0.919	0.846	0.0486
S3VM	0.854	0.785	0.818	0.761	NA
PPPredSS	**0.914**	0.962	**0.938**	**0.881**	-

The *p* values are computed between PPPredSS and others. The *p* value for S3VM was not computed

### Analysis of false positives predicted by PPPredSS

Table 4 shows the top-five false positives predicted by PPPredSS. We observed that most of the false positive sentences conveyed relationships between multiple proteins or phenotypes. It is likely difficult for the model to understand which specific relation is in focus. This issue can potentially be solved by combining all the relationships extracted from one sentence and defining linguistic patterns to find the exact relation.

**Table 4:** Top-five false positives of our proposed model

Sentence	Protein	Phenotype
“Heterozygous PU.1 mutations were reported in some patients with PHENO (AML), but not in AML with translocation t(8;21), which gives rise to the fusion gene PROT-ETO”	AML1	Acute myeloid leukemia
“PROT is also involved in the proteolytic breakdown of the extracellular matrix in PCa tumorigenesis, which contributes to tumor invasion and metastasis, and high serum PSA correlates with mutations in p53 and the overexpression of the B-cell lymphoma 2 protein, which inhibits apoptosis in PHENO cells”	PSA	Tumor
“This spectrum of somatic mutation differed from PROT mutations identified in human peripheral blood T lymphocytes and from germ-line HPRT mutations identified in Lesch-Nyhan syndrome or PHENO patients”	HPRT	Hyperuricemia
“However, a recent study, in PHENO cells, has demonstrated the involvement of p27 (increase of expression) rather than cyclin D1 in G1 cell cycle arrest induced by tunicamycin and another study, in human breast cancer cells, showed that knockdown of PROT, results in cell cycle arrest in G2/M phase”	PERK	Melanoma
“The disease is characterized by two major sets of defects; i.e., systemic purine metabolism expressed as hyperuricemia, gouty arthritis and PHENO, and dysfunction of basal ganglia and other neural pathways associated with the hallmark biochemical defect in HPRT deficiency; i.e., markedly reduced neurotransmitter dopamine (DA) in the basal ganglia in both the human and mouse PROT-deficient brain and resulting dystonia”	HPRT	Renal calculi

### Training time

All experiments were performed on a GPU system with a Tesla V100 graphics card. The fastest model is the CNN model, which took 90 seconds for training. The RNN model took 4 min for training, whereas fine-tuning the BERT model needed 10 min. In addition, since DeepPPPred utilizes the same networks with an overhead of 2 min, its training time is 17 min. The training time of PPPred is 4 min. The slowest model is S3VM, which took 160 min for training on a computer with 24 CPU cores.

### Demo curation assistant system powered by PPPredSS

Using PPPredSS as the underlying engine, we developed an in-house demo curator assistant system capable of providing the most relevant sentences for a given input. This exercise aimed to evaluate the effectiveness of PPPredSS in a real-life task; hence, we requested four biologists to test the output of this system. Our demo system’s input can be a protein name, a phenotype name, or a pair of them. For example, if the input to the system is “breast cancer”, it returns the most relevant sentences to “breast cancer.” But it can also be used to obtain a list of sentences related to a pair, e.g. “pneumonia” and “enhancer-binding protein alpha.” The predicted sentences are sorted according to the descending order of PPPredSS confidence scores, which are the average scores of probabilities output by the constituent CNN and RNN models for each class. The user had the option to adjust the number of retrieved sentences that are displayed.

When a user fed a pair composed of a protein name and a phenotype name to the demo system, it first found all the sentences stored in ProPheno [16] that co-mentions the input pair. PPPredSS generated a confidence score for each sentence in this list. These confidence scores were then used to rank the sentences. A higher confidence score indicated a higher chance that a sentence conveyed a relation between the two entities in question. Top-k sentences along with their publication venues and dates were displayed to the user. This complete process took three seconds on average for returning the ranked sentences.

### Case study: *BRCA2*-breast cancer

Table 5 shows the output (top-5 sentences) of the demo system for a well-known input pair of a human protein and an HPO term. The input protein is BRCA2 that has been mentioned in various studies for its effect on breast cancer [62, 63]. We observe that all of the top-5 returning sentences convey valid relationships between BRCA2 and breast cancer according to the column “Curator”, which reports the biologists’ manual validation. While this is not surprising given that this specific protein is well-known to be associated with breast cancer, this observation still verifies the ability of PPPredSS.

**Table 5:** The output of demo system for a well-known pair of human protein and HPO term, i.e. BRCA2 and Breast cancer

Sentence	PubMed ID	Year	Curator
“BRCA2, also known as FANCD1, is the most known gene that causes FA when both alleles are mutated and is associated with breast cancer risk when one allele is disrupted”	24765528	2014	Related
“Even more prominently, inactivation of the distal FA pathway through mutations in the BRCA2 (FANCD1) gene has been reported in breast cancer [14] (familial cases [15-17]), pancreatic cancer [18, 19] and ovarian cancer [20], among others”	26843614	2016	Related
“Our results rule out a major role of FANCI, FANCL and FANCM in familial breast cancer susceptibility, suggesting that among the 13 known FA genes, only FANCD1/BRCA2 plays a major role in high-risk breast cancer predisposition”	19737859	2009	Related
“In addition, FANCD1 gene has been shown to be identical to BRCA2, one of the two breast cancer susceptibility genes”	16115458	2005	Related
“Specifically, mutations in FANCD1 (BRCA2) carry an 82% lifetime risk of breast cancer, and 23% risk of ovarian cancer [24, 25]”	28157704	2017	Related

### Case study: *LMP7*-Hepatitis

Then we evaluated PPPredSS on a more challenging task. Table 6 demonstrates the output of the demo system for an input pair of a human protein and an HPO term that is not available in the HPO database at the time of experiments. In other words, the HPO database did not report any association between these two entities. This pair is LMP7 (“Proteasome subunit beta type-8”), and Hepatitis. According to the biologists’ manual validation, four of the top-5 sentences returned by PPPredSS conveyed valid relationships between the protein and the HPO term. This observation suggested that PPPredSS could help retrieve relevant co-mentions of pairs of entities that are not well-studied. It also indicated that the information obtained using PPPredSS could be utilized to expand the Human Phenotype Ontology database and expedite the process by assisting curators. Note that the second retrieved sentence, which is the title of an article, does not explicitly convey a relation by itself (and hence labeled as “invalid” by the biologists). But note that the corresponding article does contain evidence of a valid relationship.

**Table 6:** The output of demo system for a pair of human protein and HPO term, i.e. LMP7 and Hepatitis, that is less well-known

Sentence	PubMed ID	Year	Curator
“Other reports have revealed that LMP2/LMP7 genes are strongly correlated with the hepatitis B infection[12],[13]”	23554652	2010	Related
“[Association between LMP2/LMP7 gene polymorphism and the infection of hepatitis B virus]”	16224524	2005	Invalid
“One report from Japan revealed that LMP7-145 SNP is one of the important host factors which independently influences the response to IFN in patients with chronic hepatitis C[18]”	23554652	2010	Related
“Hepatitis C virus non-structural protein NS3 interacts with LMP7, a component of the immunoproteasome, and affects its proteasome activity”	15303969	2004	Related
“These findings suggest that a single nucleotide polymorphism of LMP7 gene is one of the important host factors which independently influence the response to IFN in patients with chronic hepatitis C”	12225333	2002	Related

### General-purpose Search vs. PPPredSS

Biologists and other researchers typically end up using general-purpose search engines such as Google^6^ for document triage due to the lack of domain-specific search engines for biological entities such as proteins and phenotypes. Therefore, we compare the output of our demo system with the Google search engine results for the same entity pairs to highlight the utility of PPPredSS.

**Fig. 3:**
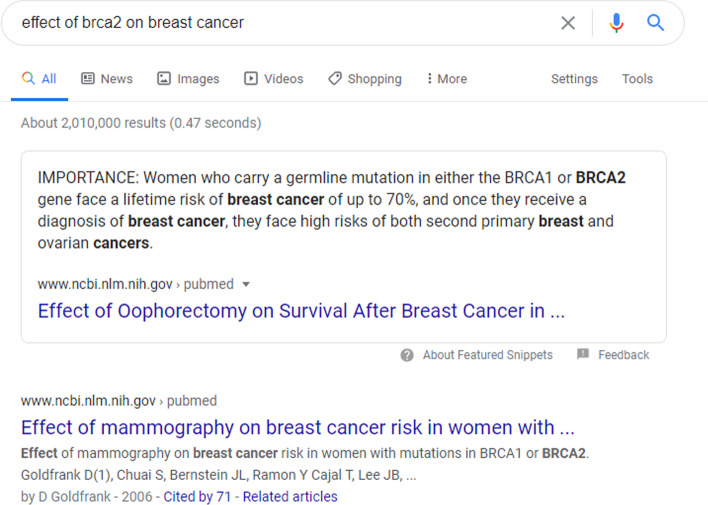
The output of Google search engine with the query “effect of BRCA2 on breast cancer”. Google extracted a text span that includes information about the pair. This search was performed on June 20, 2020 at 11:18 PM MST

When the query “Effect of BRCA2 on breast cancer” was fed to the Google search engine, it returned a list of “hits.” It also returned the following text span that expresses a relationship between the input protein and phenotype: “Women who carry a germline mutation in either the BRCA1 or BRCA2 gene face a lifetime risk of breast cancer of up to 70%, and once they receive a diagnosis of breast cancer, they face high risks of both second primary breast and ovarian cancers.” (see Fig. 3).

**Fig. 4:**
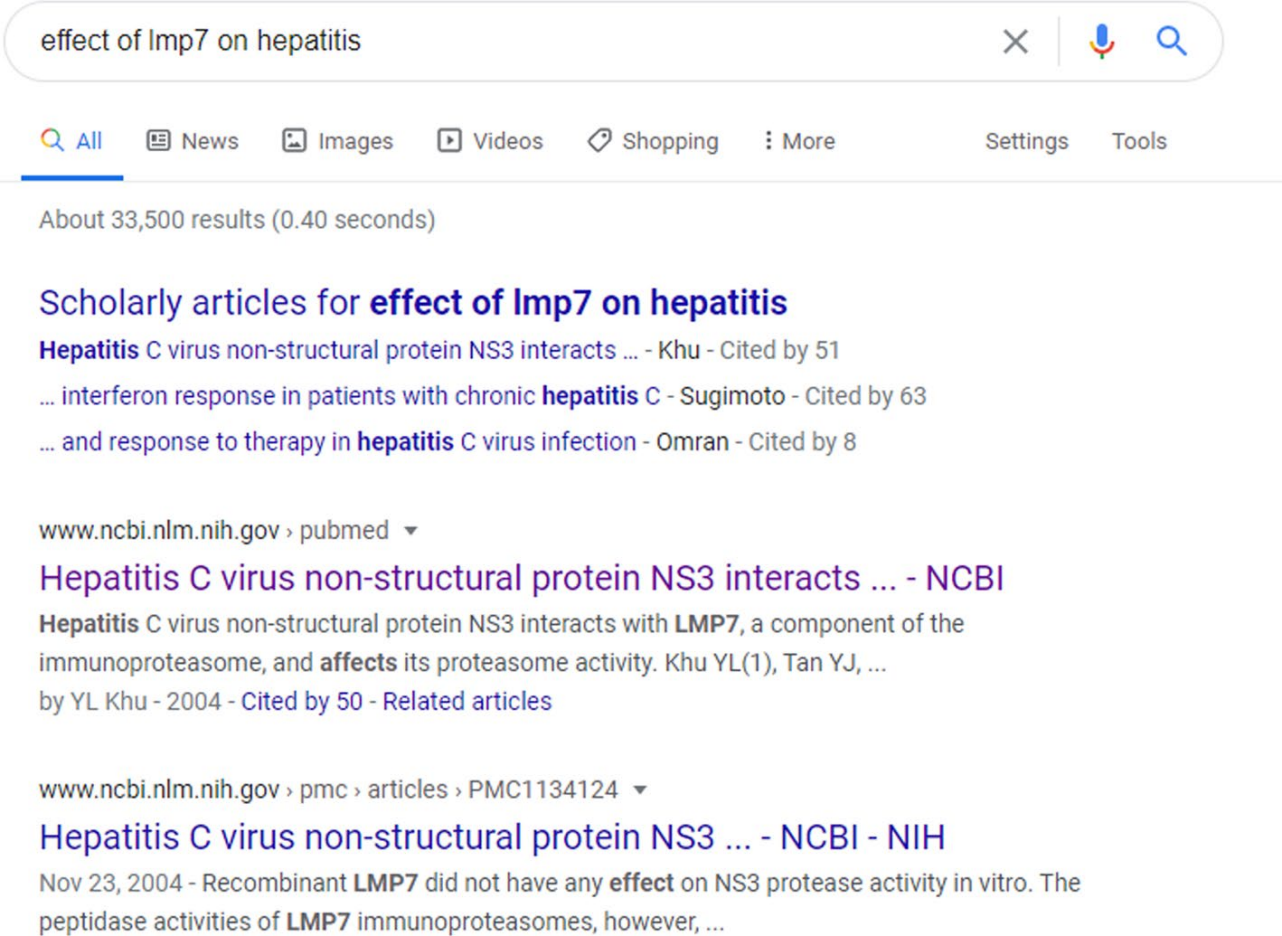
The output of Google search engine with the query “effect of LMP7 on Hepatitis”. Google returns a list of published papers that include the input pair. This search was performed on June 20, 2020 at 11:25 PM MST

Similarly, we also obtained the output of Google for the pair of LMP7 and Hepatitis. However, as shown in Fig. 4, by feeding the query “Effect of LMP7 on Hepatitis”, only a list of articles was displayed (i.e., Google did not extract a relevant text span as in the previous example). This observation suggested that while Google may help with well-known pairs of entities, it may be inadequate for other challenging queries. In this situation, the user must manually read through the articles or, at the very minimum, read the abstracts of articles to acquire the desired information about the relationship between the pair, which could be time-consuming. However, our demo system powered by PPPredSS simplifies this process by immediately returning only the sentences containing a relationship between the protein and phenotype. The users can refer to the corresponding published articles only if they need additional information beyond what is mentioned in the sentences.

### Biologists’ feedback

We wanted to understand how a typical researcher/biologist queries for biological entities of interest and how their experience of using a general-purpose search engine compares to our curation assistant system (powered by PPPredSS). So, we asked four biologists to query for the above two entity pairs (i.e., BRCA2-breast cancer and LMP7-Hepatitis) using Google. In other words, we requested them to find evidence that the two pairs had relationships without restricting how they perform the tasks (i.e., no restriction on the number of searches/queries or the amount of reading). As a comparison, we provided them the top-5 sentences retrieved by PPPredSS for the same pairs of entities (shown in Tables 5, 6 ) and was asked whether and how PPPredSS sentences enrich their experience. Furthermore, we created a questionnaire to understand and describe their process completely. Their complete responses to the questionnaire are given in the Appendix. The summary of their feedback is shown in Tables 7 and 8 for BRCA2 and LMP7, respectively. The consensus was that the four biologists found PPPredSS-based output more convenient and informative than Google output, especially for the less-well-studied pair of entities (i.e., LMP7-Hepatitis).

**Table 7:** Four biologists’ experience with finding a relationship between BRCA2 and Breast Cancer using Google and how it compares to PPPredSS retrieved sentences

BRCA2 and breast cancer				
	Biologist 1	Biologist 2	Biologist 3	Biologist 4
# of Searches	1	1	1	1
Keywords	brca2 breast cancer association	The relationship between BRCA2 and breast cancer	brac2 breast cancer	brca2 breast cancer
Amount of reading	A few sentences	Reading highlighted text by Google	One sentence	Skimming the first page of results
Duration	1 min	1 min	1 min	1 min
Winner	Tie	PPPredSS	PPPredSS	PPPredSS

# of Searches: number of queries used, Keywords: queries used for each search, Amount of reading: length of the text read, Winner: the tool that provides better results out of Google and PPPredSS

In summary, the above case studies demonstrate the ability of PPPredSS to facilitate expediting the biocuration process by extracting the most relevant sentences on human protein-phenotype pairs. They also exemplify how it can be integrated into a curation pipeline employed by bio-curators to expand knowledge bases and ontologies such as HPO. Even though the accuracy of PPPredSS is very high, we are in no way suggesting that the curation process should or can be fully automated. The process of biocuration of HPO, or any other knowledge base, is highly nuanced and involved than merely finding relevant sentences. Therefore, we recommend our model to be a complementary tool for bio-curators to expedite the process by prioritizing which articles to dig deeper into.

## Conclusion

This work proposes a novel deep semi-supervised ensemble framework to classify sentence-level co-mentions of proteins and phenotypic abnormalities associated with human diseases. Our framework’s inputs are a corpus of biomedical articles, a list of protein and phenotype names, and a small labeled dataset of sentences. First, it extracts the complete list of sentences containing protein-phenotype co-mentions from biomedical articles. Then, it trains a supervised classifier on the small labeled dataset. Next, using the trained model, it predicts the labels for unlabeled sentences. It then expands the training set and increases the number of labeled instances by picking a subset of top predictions. Eventually, using an ensemble of deep learning classifiers provides a more robust model that gives accurate predictions on unseen pairs of entities. This framework can return a list of the most relevant sentences for a given pair of a protein and a phenotype with their corresponding confidence scores.

We developed a prototype of our framework, PPPredSS, that used BERT as the first supervised classifier and utilized a combination of RNNs and CNNs as the ensemble classifier. Our experimental results demonstrated that PPPredSS provides excellent performance compared to fully-supervised models such as PPPred and DeepPPPred. It also significantly outperformed S3VM (the state-of-the-art Semi-supervised SVM) trained using around one million additional instances. We further developed an in-house demo curation assistant system powered by PPPredSS and analyzed its output for two case studies compared to a general-purpose search engine. Feedback from the group of biologists on these outputs further highlights the utility of PPPredSS.

While PPPredSS is very accurate, there are many different avenues for future research. The accuracy of the named entity recognizer tools directly affects the quality of our framework. Our dataset of entities lacks some proteins and phenotypes due to errors in upstream named entity recognition tools. In other words, our dataset covers 2512 unique proteins and 2277 unique phenotype names compared to 4589 and 9795 proteins and phenotypes currently curated in the official HPO database, respectively. One of the next steps is to investigate a plethora of entity recognition tools to improve the overall coverage of PPPredSS. Also, while BERT is very accurate, it is still costly to fine-tune BERT. Therefore, utilizing lighter models such as ALBERT is a potential future work. Another possible future work is to replace BERT with BioBERT [64] (i.e., a BERT model pre-trained on biomedical text), which would likely improve the overall performance.

Another limitation of PPPredSS is that it is restricted to sentence-level co-mentions. However, it is known that 10–15% valid relationships are expressed between entities mentioned across sentence boundaries. Therefore, we plan to investigate incorporating paragraph-level co-mentions while still maintaining similar runtimes. Finally, We would also like to develop a public interactive web-server powered by PPPredSS that can be used by both biocurators and researchers working in this area. A diverse collection of case studies including unclear/controversial proteins and phenotypes pairs would provide valuable feedback for setting up such a system.

**Table 8:** Four biologists’ experience with finding a relationship between LMP7 and Hepatitis using Google and how it compares to PPPredSS retrieved sentences

LMP7 and hepatitis				
	Biologist 1	Biologist 2	Biologist 3	Biologist 4
# of Searches	5	1	1	1
Keywords	LMP7 and hepatitis, LMP7 and hepatitis association, LMP7, What is LMP7	The relationship between LMP7 and hepatitis	lmp7 hepatitis	LMP7 and Hepatitis
Amount of reading	A few minutes of reading	Reading the conclusions of one paper	One sentence	Skimming the first page of results
Duration	5 min	3 min	1 min	2 min
Winner	PPPredSS	PPPredSS	Tie	PPPredSS

# of Searches: number of queries used, Keywords: queries used for each search, Amount of reading: length of the text read, Winner: the tool that provides better results out of Google and PPPredSS

## Methods

### Approach

**Fig. 5:**
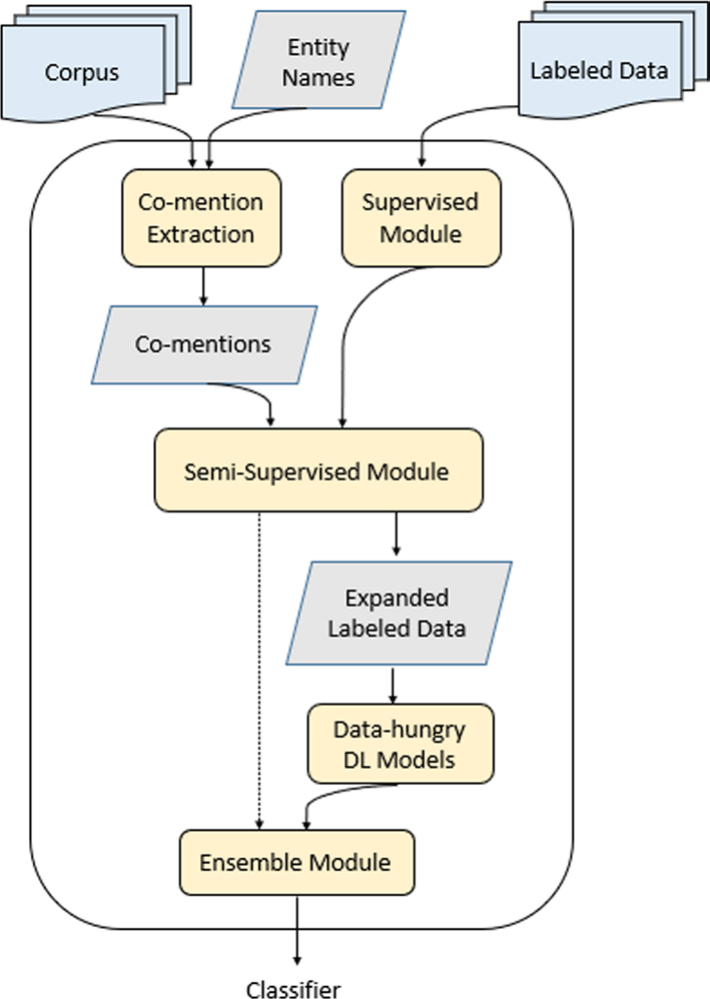
The proposed framework that combines supervised learning, semi-supervised learning, and ensemble learning

Our proposed framework is a combination of semi-supervised learning, deep learning, and ensemble learning. Figure 5 depicts the proposed framework. The inputs to this framework are (1) a small labeled dataset composed of labeled protein-phenotype co-mentions, (2) the entire corpora of biomedical articles, and (3) the names of proteins and phenotypes.

First, we extracted an unlabeled list of sentences that contain a protein and a phenotype name from the biomedical articles. The “Co-mention Extraction” module takes a corpus and a list of entities as input and returns text spans, e.g., sentences, which contain a co-mention of the entities of interest as output.

The supervised module is trained on labeled data and is capable of generating labels for unlabeled instances. Using a supervised learning algorithm, we trained a model on the labeled dataset and made predictions on the unlabeled dataset. There are multiple options for the supervised module, including BERT, SVM, etc.

Semi-supervised learning helps increase the training set size by combining the labeled instances with the unlabeled dataset predictions. It can achieve this using self-training, co-training, or other approaches. The semi-supervised module takes a trained supervised module and a list of text spans containing the entities of interest as input. It returns labels for the unlabeled text spans as output. This module is also capable of improving itself in the training process.

The high-confidence predictions made by the semi-supervised module are added to the labeled data to expand it. After growing the labeled data, we had access to enough data for the data-hungry deep learning models, e.g., CNNs, RNNs, etc. Therefore, we trained multiple deep learning models on the expanded labeled data.

Next, we created an ensemble module by combining the deep learning models. The ensemble model can be obtained using either averaging or stacking. This framework’s final output is a classifier capable of classifying text spans composed of entities of interest into either positive or negative classes.

This framework only requires a corpus, a list of entities, and a relatively small labeled dataset. It is independent of the type of entities and corpus. So, in theory, it can be utilized for any task of binary relation extraction in other domains as long as the required three input data sources are available for those domains. The minimum number of labeled instances depends on the complexity of the domain and can be determined experimentally.

### Data

We used the entire collection of Medline abstracts (downloaded on 07/01/2017) and PMC full-text articles (downloaded on 03/15/2018) as mentioned in [16]) as our corpus. The names of proteins and phenotypes are fed using their corresponding UniProt IDs and HPO IDs, respectively. We employed the gold-standard dataset that we created in our previous study [17], which comprises 1,685 co-mentions annotated by biologists. The gold-standard dataset is split into train, validation, and test data using random stratification. The number of sentence-level co-mentions in the training, validation, and test sets are 1010 (60%), 337 (20%), and 337 (20%). The training, validation, and test sets remain unchanged during training and inference.

### Preprocessing

Because there are multi-word protein and phenotype names, we first replaced all the protein and phenotype entities in the sentences with “PROT” and “PHENO,” respectively. Subsequently, we utilized tokenization to break the unstructured text into a list of words/phrases and converted them into a list of numerical sequences understood by our models. Next, we truncated/padded the sequences that are longer/shorter than a threshold to ensure the same dimensionality across all the sentences.

### Models

As mentioned before, we implemented PPPredSS as a prototype of the proposed framework. We provided the unlabeled co-mentions extracted from ProPheno [16] and the small labeled dataset (i.e., gold-standard co-mentions) as input, and we trained PPPredSS using the algorithm given in Algorithm 1.

We first fine-tuned the BERT model on our small labeled dataset. BERT provides very accurate predictions when trained on labeled sentences. Therefore, by allowing it to make predictions on the unlabeled sentences, we obtained high-quality predictions on millions of unlabeled instances. Using the validation set, we iteratively looked for the subset of such prediction that improves the BERT model’s overall accuracy. Next, we added this subset of top predictions to the training set and obtained an expanded training set. Details of pre-training and fine-tuning the BERT model are described elsewhere [17]. 
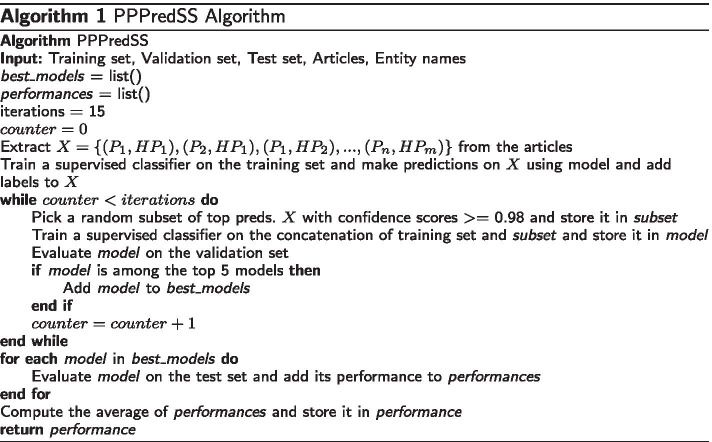


The expanded training set is fed to a model that is composed of RNNs and CNNs. In our previous study [17], we showed that CNNs perform better on shorter sentences, whereas RNNs provide better performance on longer sentences. Therefore, we employed the same CNN and RNN architectures in the current study and averaged their predictions to develop the ensemble model. These RNN and CNN models generate confidence scores, which is the average of probabilities returned by the RNN and CNN models for each instance in the validation set. We computed the average of each instance’s confidence scores and reported it as the instance’s final confidence score. Model architectures and details of training individual RNN and CNN models are described elsewhere [17]. Finally, we used this ensemble model to make predictions on the test data to evaluate PPPredSS and compared it to other competitors.

### Experimental setup

We used PyTorch^7^ and SciKit-learn^8^ packages for our implementations. We trained the CNN and RNN models for 20 epochs. We used the binary cross-entropy loss as the loss function and the Adam optimizer as the optimizer. The BERT model is fine-tuned in four epochs. All of these parameter values were obtained experimentally based on the validation accuracy.

We used various random seeds to perform 10-times hold-out validation (i.e., the same split with different seeds was used for repeats). Next, we averaged them to compare the performance of the presented model with other baseline models. Precision, recall, and F1 metrics were used as the primary performance metrics. The formal definitions of those metrics are given below. We also report area under the receiver operating characteristics curve (AUROC) [65] values. Finally, we used paired t-tests to measure the significance of the performance differences across different models.

$$\begin{aligned} Precision&= \frac{\text {True Positives}}{\text {True Positives} + \text {False Positives}}\\ Recall&= \frac{\text {True Positives}}{\text {True Positives} + \text {False Negatives}}\\ F1&= \frac{2 * Precision * Recall}{Precision + Recall} \end{aligned}$$

## Data Availability

The datasets generated and analyzed during the current study are available in the Zenodo repository, http://doi.org/10.5281/zenodo.3965127. The software/scripts are available at https://doi.org/10.5281/zenodo.4568364. The software for DeepPPPred, which includes the script for PPPred, is also available at https://github.com/mpourreza/DeepPPPred.

## Appendix

This section provides the questionnaire used to acquire the biologists’ feedback on our in-house demo curation assistant system powered by PPPredSS.

- Question 1: how many Google searches did you perform to get the desired information?
- Question 2: What were the keywords that you used for the searches?
- Question 3: Did Google pop up the answer to your input search query or you had to open the page and scan through the document?
- Question 4: How much did you have to read to get the answer?
- Question 5: How long did it take for you to get the answer?
- Question 6: How do you compare the results from Google search with the sentences provided by us?

The following are the responses by the four biologists.

### Biologist 1 (Katrina Lyon)

#### BRCA2 and breast cancer

1. Google searches to get desired information: 1
2. Keywords used: “brca2 breast cancer association”
3. A google search result directly identified BRCA as “the breast cancer gene”.
4. A few sentences, maximum.
5. Found the answer in less than a minute from nationalbreastcancer.org.
6. The generated sentences largely describe BRCA2 in the context of both breast cancer and Fanconi anemia. Fanconi anemia did not pop up in the search results. It appears that the predictor pulled these sentences from literature comparing breast cancer susceptibility to BRCA2/FANCD1 expression. The generated sentences did not go farther than indicating an association between breast cancer risk and BRCA2.

#### LMP7 and hepatitis

1. Google searches to get desired information: 5
2. Keywords used: “LMP7 and hepatitis” “LMP7 and hepatitis association” “LMP7” “What is LMP7”
3. My google search results did not provide a direct answer as to the relationship between LMP7 and Hepatitis, so I had to do some scanning through the literature.
4. A few minutes of reading were enough to find the relationship.
5. Approximately 5 min.
6. Sentences provided went into far more detail than the google search results yielded. The sentences were also more helpful in determining the relationship/extent thereof between LMP7 and Hepatitis.

### Biologist 2 (Julia Schearer)

1. Question 1: how many Google searches did you perform to get the desired information? One Google search for each.
2. Question 2: What were the keywords that you used for the searches? The relationship between BRCA2 and breast cancer. The relationship between LMP7 and Hepatitis.
3. Question 3: Did Google pop up the answer to your input search query or you had to open the page and scan through the document? For BRCA2 and breast cancer, Google popped up the answer to the search query. Shown below. For LMP7 and Hepatitis, I had to open a scientific journal article and scan through a document to find the answer.
4. Question 4: How much did you have to read to get the answer? For BRCA2 and breast cancer, the answer popped up right away and therefore I just had to read that. I have read many journal articles and therefore have become efficient at finding answers in these papers. I scrolled down to the discussion/conclusions of the paper and found the relationship between LMP7 and Hepatitis by just reading these sections.
5. Question 5: How long did it take for you to get the answer? 30 seconds for BRCA2 and breast cancer. 3 min for LMP7 and Hepatitis.
6. Question 6: How do you compare the results from Google search with the sentences provided by us? The sentences that were provided by you all were very helpful in showing the association, as well as providing more detail about how this association occurs. On Google, usually, I would search something and find that there was an association between the two, but then have to do more searches or reading to find out how exactly they are related to one another.

### Biologist 3 (Mandi M. Roe)

#### BRCA2/breast cancer

1. Q1: One google search to get the desired information.
2. Q2: I used the keywords: BRCA2 breast cancer
3. Q3: Google popped up with the answer of my research inquiry and answered the question without me having to open the page. It was from the CDC website.
4. Q4: I had to read one sentence to get the answer to the relationship between BRCA2 and breast cancer.
5. Q5: It took me less than 1 min to get the answer.
6. Q6: The results that I got from my google search did not include any information about FA or the connection between FANCD1 and BRCA2. The results I found were only associated with the two input words I put into the search.

#### LMP7/hepatitis

1. Q1: One google search to get the desired information
2. Q2: I used the keywords: LMP7 hepatitis
3. Q3: Google did not pop up with the answer to my inquiry, however, I did not have to open a page to get the answer to my question. The first search result was a paper and the sentences beneath the title described hepatitis virus and LMP7, as well as, explaining interactions between the two.
4. Q4: I had to read one sentence to get the answer to the relationship between LMP7 and Hepatitis.
5. Q5: It took me less than 1 min to get the answer.
6. Q6: The sentence I read that was at the top of my google search was the same as sentence 4 in the LMP7/hepatitis sentences you provided.

### Biologist 4 (Gillian Reynolds)

1. Question 1: how many Google searches did you perform to get the desired information? BRCA2 and breast cancer - just a single google search is adequate to find information geared towards the general public and even some scientific results. It’s a very high profile gene so this is unsurprising. LMP7 and Hepatitis - just a single google search is adequate to find scientific information of their association.
2. Question 2: What were the keywords that you used for the searches? I simply used “brca2 breast cancer” and “LMP7 and Hepatitis”.
3. Question 3: Did Google pop up the answer to your input search query or you had to open the page and scan through the document? Google popped up enough information for me to get the general gist for both searches. If more detail/specifics is required then I’d have to search through the articles.
4. Question 4: How much did you have to read to get the answer? I was able to skim the first page of results to get an understanding of their relationship.
5. Question 5: How long did it take for you to get the answer? A couple of minutes per search.
6. Question 6: How do you compare the results from Google search with the sentences provided by us? The google searches provide me some information on the protein/disease association, your sentences provide me more specific details of that relationship.

## Abbreviations

AUROC: Area under the receiver operator characteristics curve
BERT: Bidirectional encoder representations from transformers
CNN: Convolutional neural network
DAG: Directed acyclic graph
HPO: Human phenotype ontology
RNN: Recurrent neural network
SVM: Support vector machines
S3VM: Semi-supervised support vector machines

## Footnotes

1 https://hpo.jax.org/app.

2 http://propheno.cs.montana.edu.

3 http://doi.org/10.5281/zenodo.3965127.

4 https://doi.org/10.5281/zenodo.4568364.

5 https://biocreative.bioinformatics.udel.edu/tasks/biocreative-vi/track-5/.

6 https://www.google.com/.

7 https://pytorch.org.

8 https://scikit-learn.org.
